# D-Amphetamine Accelerates Recovery of Consciousness and Respiratory Drive After High-Dose Fentanyl in Rats

**DOI:** 10.3389/fphar.2020.585356

**Published:** 2020-11-12

**Authors:** Olivia A. Moody, Edlyn R. Zhang, Vipin Arora, Risako Kato, Joseph F. Cotten, Ken Solt

**Affiliations:** ^1^ Department of Anesthesia, Critical Care and Pain Medicine, Massachusetts General Hospital, Boston, MA, United States; ^2^ Department of Anaesthesia, Harvard Medical School, Boston, MA, United States

**Keywords:** d-amphetamine, respiratory depression, arterial blood gas, fentanyl, anesthesia, neuroscience

## Abstract

In the United States, fentanyl causes approximately 60,000 drug overdose deaths each year. Fentanyl is also frequently administered as an analgesic in the perioperative setting, where respiratory depression remains a common clinical problem. Naloxone is an efficacious opioid antagonist, but it possesses a short half-life and undesirable side effects. This study was conducted to test the hypothesis that d-amphetamine ameliorates respiratory depression and hastens the return of consciousness following high-dose fentanyl. Behavioral endpoints (first head movement, two paws down, and return of righting), arterial blood gas analysis and local field potential recordings from the prefrontal cortex were conducted in adult rats after intravenous administration of of fentanyl (55 µg/kg) at a dose sufficient to induce loss of righting and respiratory depression, followed by intravenous d-amphetamine (3 mg/kg) or saline (vehicle). D-amphetamine accelerated the time to return of righting by 36.6% compared to saline controls. D-amphetamine also hastened recovery of arterial pH, and the partial pressure of CO2, O2 and sO2 compared to controls, with statistically significant differences in pH after 5 min and 15 min. Local field potential recordings from the prefrontal cortex showed that within 5 min of d-amphetamine administration, the elevated broadband power <20 Hz produced by fentanyl had returned to awake baseline levels, consistent with the return of consciousness. Overall, d-amphetamine attenuated respiratory acidosis, increased arterial oxygenation, and accelerated the return of consciousness in the setting of fentanyl intoxication. This suggests that d-amphetamine may be a useful adjunct or alternative to opioid receptor antagonists such as naloxone.

## Introduction

Fentanyl is 50–100 times more potent than morphine (Suzuki and El-Haddad, 2017), and causes respiratory depression, chest wall rigidity, and loss of consciousness which can rapidly lead to hypoxia, hypercarbia, and death if not treated promptly (Henderson et al., 2014; Lalley et al., 2014; Torralva and Janowsky, 2019). Fentanyl is commonly administered as a potent analgesic in a variety of formulations, including intravenous, transdermal, and sublingual. Although intravenous fentanyl administration is generally safe in the perioperative setting with appropriate monitoring, opioid-induced respiratory depression is still a common postoperative complication that can affect up to 17% of patients (Cashman and Dolin, 2004). In addition, fentanyl causes approximately 60,000 drug overdose deaths each year in the United States, making it a significant public health problem (Scholl et al., 2018; Torralva and Janowsky, 2019).

The µ opioid receptor antagonist naloxone is the mainstay for treating opioid overdoses in the hospital and community settings, but it has several major limitations. In the clinical setting, where fentanyl is often administered to manage severe pain, naloxone not only reverses respiratory depression but also desired analgesia (Rzasa Lynn and Galinkin, 2018). In the community setting, naloxone can precipitate painful withdrawal symptoms in people with opioid use disorder (Rzasa Lynn and Galinkin, 2018). Naloxone can also produce rare but life-threatening side effects, such as pulmonary edema, cardiac arrhythmias, seizures, and cardiac arrest (Dahan et al., 2010). Finally, naloxone has a short plasma half-life and rapid pharmacokinetics, making it necessary to administer repeated doses or a continuous intravenous infusion to maintain antagonism of opioids with slower pharmacokinetics (Dahan et al., 2010; Suzuki and El-Haddad, 2017). Therefore, there is an unmet need for alternatives to naloxone that provide respiratory stimulation and restoration of consciousness with fewer side effects.

While reversal of respiratory depression is essential after a fentanyl overdose, restoring consciousness is also critically important. Conscious patients are likely to respond to verbal reminders to breathe – this is commonly observed in the operating room when patients receive fentanyl at doses sufficient to produce respiratory depression but not unconsciousness. Therefore, an ideal fentanyl reversal agent would reverse both respiratory depression and unconsciousness. In a previous study, d-amphetamine was shown to restore consciousness in rats under general anesthesia with propofol or sevoflurane, an effect that was primarily driven by dopamine type 1 (D_1_) receptors (Kenny et al., 2015). Another study found that a D_1_ receptor agonist attenuates fentanyl-induced respiratory depression without affecting analgesia in cats (Lalley, 2004, 2005). D-amphetamine increases the extracellular levels of dopamine, norepinephrine and serotonin in the brain by blocking and reversing the presynaptic uptake of monoamines and acting as a monoamine oxidase inhibitor (Heal et al., 2013). All of these neurotransmitters are known to increase wakefulness (Brown et al., 2011).

D-amphetamine is widely prescribed in oral form for the treatment of Attention Deficit Hyperactivity Disorder, and is known to be safe and well-tolerated in humans (Heal et al., 2013). However, d-amphetamine has not been tested previously as a potential treatment for fentanyl overdose. In this study, behavioral assessments, arterial blood gas analysis, and neurophysiological recordings were conducted in rats to test the hypothesis that d-amphetamine reverses fentanyl-induced respiratory depression and loss of consciousness.

## Materials and Methods

### Animals

All studies were conducted in accordance with the recommendations from The Guide for the Care and Use of Laboratory Animals, National Institutes of Health and approved by the Massachusetts General Hospital Institutional Animal Care and Use Committee. Reporting of *in vivo* animal research in this study complies with the ARRIVE guidelines (Kilkenny et al., 2010).

Adult male and female (n = 8 each) Sprague-Dawley rats (Charles River Laboratories, Wilmington, MA) were used for all behavioral and blood gas experiments. Animals had chronic femoral artery catheters that were implanted by Charles River Laboratories prior to arrival at the animal care facility. Animals were individually housed on a standard 12-h light schedule (lights on at 7:00 am, lights off at 7:00 pm) with *ad libitum* access to food and water. Weights ranged from 285–490 g at the time of the experiments. Animals were habituated to handling at least 3 days prior to the start of experiments and all experiments were conducted between 9 am and 3 pm in the animals’ home cage. A heating pad placed under the cage was used to maintain body temperature at 37°C during experiments. A minimum of 7 days of rest was provided between experiments for all animals.

To determine the group sample sizes, a pilot trial (n = 8 rats) assessed the time to ROR after fentanyl when saline vs. d-amphetamine was administered. D-amphetamine shortened time to ROR by 60.4% with an effect size of 1.6. Using G*Power (G*Power, Heinrich-Heine-Universität Düsseldor, Germany) (Faul et al., 2007), we predicted a sample size of six rats would give 0.80 power (β) when α = 0.05 for a two-way t test (t (5) = 2.57). Although predicting effect size from smaller pilot studies has its limitations, our initial pilot study (n = 8 rats per group) showed a robust effect of d-amphetamine compared to saline. Subsequently, eight new male and eight new female rats were used for this study to allow for the detection of potential sex differences, and to ensure group sizes ≥6 in case the arterial catheters failed in up to 25% of animals.

**FIGURE 1:**
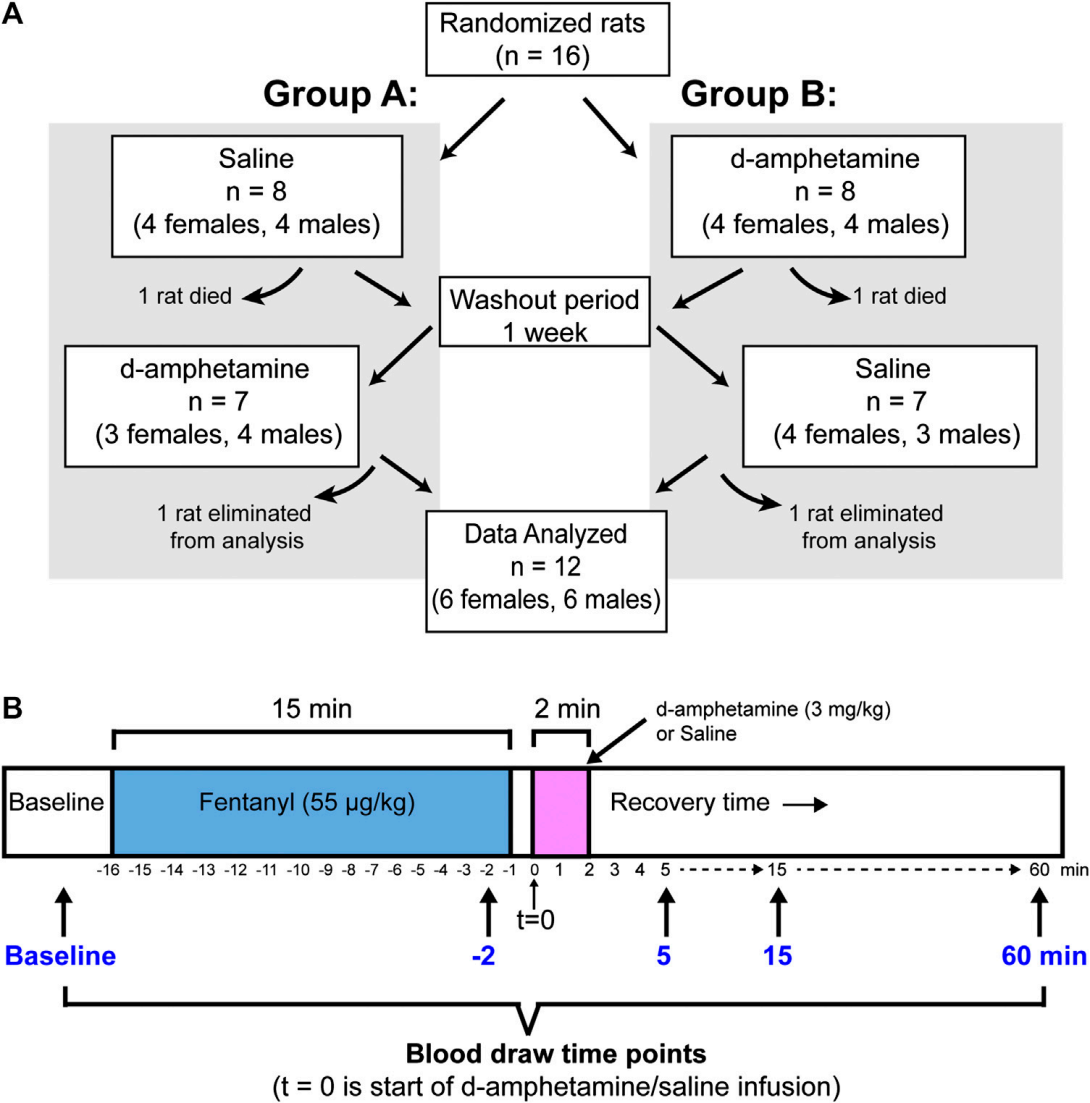
The experimental design for animal assignment and drug administration. **(A)** A blinded crossover design was used to randomly assign rats to treatment groups (Group A: saline first; Group B: d-amphetamine first). After experiment 1, the rats received a 1-week rest period and then received the opposite treatment (d-amphetamine or saline). Final datasets for behavioral or arterial blood gas analysis were only included if animals completed the entire protocol. **(B)** A timeline showing the protocol for an individual experiment. Fentanyl (55 µg/kg, IV) was administered over 15 min and then d-amphetamine (3 mg/kg, IV) or saline (vehicle) was administered over 2 min. There was 1 min between fentanyl and d-amphetamine infusions to allow for the exchange of drug lines. Arterial blood was drawn for blood gas analysis before the experiment (baseline), 1 min before the end of the fentanyl infusion (t = −2) and 5, 15 and 60 min from the start of the d-amphetamine infusion. All behavioral and blood gas time points were referenced to the start of the d-amphetamine or saline infusion (t = 0).

A blinded crossover design was used to assign animals to d-amphetamine and saline treatment groups (Figure 1A). The advantages of a crossover design are that each subject acts as their own control and a smaller number of animals are needed relative to parallel-group studies. The limitations are that a washout period is needed between treatments and the study can be vulnerable to interaction effects, such as drug order (Suchmacher and Geller, 2012)., In total, 16 rats (eight males and eight females) were initially included in the behavioral and blood gas experiments detailed below. However, data was only analyzed for animals that completed both the saline and d-amphetamine experiments. Four rats were excluded from the study because two did not survive the fentanyl infusion and two had no loss of righting during the second fentanyl infusion in week two. Five additional animals were excluded from the blood gas analysis due to failed arterial catheters that prevented blood from being drawn in week two, yielding an incomplete dataset.

### Drugs

Fentanyl citrate (50 mcg/mL) was acquired from Hospira, Inc (Lake Forest, IL). Dextroamphetamine sulfate was acquired from Millipore Sigma (#1180004, Rockville, MD). All drugs were administered intravenously. Fentanyl was prepared daily by diluting it in normal saline (0.9% sodium chloride) to a final concentration of 10 µg/ml. D-amphetamine was dissolved in saline to achieve a final concentration of 1.5 mg/ml. To ensure a blinded experiment, 1.5 ml aliquots of d-amphetamine or saline were prepared 1 week ahead and coded by an independent group member and stored at −20°C until the day of use. Drugs were administered via intravenous (IV) tubing (approximately 0.5 ml) primed with each drug and attached to a syringe pump (Harvard Apparatus, Holliston, Massachusetts).

### Arterial Blood Gas Analysis

Arterial blood gas analysis was performed with an i-STAT Alinity V Analyzer (Abaxis, Union City, CA) using i-STAT CG4+ and CG8+ cartridges (Abbott, Princeton, NJ). Blood gas measurements included pH, partial pressure of carbon dioxide (PACO_2_), partial pressure of oxygen (PaO_2_), and oxygen saturation (sO_2_). At the start of the day, animals were gently restrained by hand and an initial blood sample was analyzed prior to exposure to any anesthetics. Samples were collected by withdrawing 0.1 ml from the arterial catheter and discarding it, then drawing up 0.2 ml of fresh blood using a 1 ml syringe with 2 µL of heparin at the syringe tip (500 U/ml heparin, HGS-50, SAI Infusion Technologies, Lake Villa, IL). The catheter was flushed with 0.5–1 ml of 10 U/ml heparinized saline after each use to prevent clotting. Blood was analyzed immediately at 37°C. At the end of the experiment, the catheter was flushed with a 1 ml mixture of 1:1 heparin (500 U/ml heparin) to 50% dextrose (25 g/50 ml, Hospira, Inc, Lake Forest, IL). Hemoglobin was measured at the start of each experimental day in a subset of eight animals using the CG8+ cartridge. Blood loss over the course of the experiment was estimated to be approximately 1.5 ml (approximately 8% of total blood volume for a 300 g rat). No significant decline in hemoglobin concentration was measured at the start of the second experimental day 1 week later.

### Experimental Design

A blinded crossover design was used to control for the order of d-amphetamine vs. saline and to account for the possibility of tolerance to fentanyl in week two (Figure 1A). Rats were randomly assigned to one of two groups, each with four males and four females per group (eight rats total per group). In week one, Group A received saline and Group B received d-amphetamine. In week two, Group A received d-amphetamine and Group B received saline.

On the day of the experiment, animals were anesthetized with 2–3% isoflurane (McKesson, Northborough, MA) in oxygen and underwent cannulation of the lateral tail vein for the administration of intravenous drugs. Isoflurane exposure lasted 15 min on average. Animals fully recovered from isoflurane anesthesia for at least 30 min before starting any fentanyl experiments (average time before starting was 50 min). A baseline heart rate and sO_2_% was recorded with a pulse oximeter (2500A Vet, Nonin Medical, Inc., Plymouth, NJ) during this period.

In pilot experiments using a different set of rats, intravenous doses from 50–60 µg/kg were tested based on previously published doses used in rats (Ren, Ding et al., 2009, Henderson, May et al., 2014). The intravenous dose of 55 µg/kg fentanyl infused over 15 min was selected because it reliably produced loss of righting (LOR, indicating loss of consciousness) lasting approximately 20 min with concomitant respiratory depression. In addition, this dose is similar to another study in rats that found that 60 µg/kg (IV) of fentanyl over 20 min produced robust respiratory depression but did not produce lethal apnea that a higher dose (80 µg/kg, IV) did produce (Ren, Ding et al., 2009). The d-amphetamine dose 3 mg/kg (IV) over 2 min was selected based on our previous published work measuring the dose-dependent effects d-amphetamine (0.3–3 mg/kg, IV) reversal of sevoflurane-induced unconsciousness (Kenny, Taylor et al., 2015). Since 1 and 3 mg/kg showed no significant difference in effect, the higher dose of 3 mg/kg was selected here to produce a maximal effect after a high dose of fentanyl.

At the start of the experiment, intravenous fentanyl (55 µg/kg) was administered over 15 min to rats. Pulse oximetry was recorded via a probe placed on the hind paw 12 min after the start of the fentanyl infusion (the average time at which LOR was observed) and removed when the animals had return of righting (ROR). An arterial blood sample was drawn 1 min before the end of the fentanyl infusion (t = −2 min). After the end of the fentanyl infusion, 1 min was taken to exchange drug lines before intravenous d-amphetamine (3 mg/kg) was infused over 2 min. The animals were carefully monitored in their home cages during recovery and the time to righting was recorded. Three additional arterial blood samples were drawn at 5, 15, and 60 min after the start of the d-amphetamine infusion (t = 0). Figure 1B illustrates the sequence of drug delivery and timing of blood draws for each experiment.

### Behavioral Endpoints

A continuous video recording was taken to assess timing of behavioral endpoints, and all behavioral measurements were independently scored by a researcher blinded to the drug treatment. ROR, a well-accepted surrogate measure of return of consciousness in rodents (Franks, 2008), was defined as the return of all four paws to touching the ground. Time to first head movement was scored as the time when an animal first distinctly moved its head in any direction. The time to two paws down was defined as the time when the animal had placed any two paws stably on the ground in a weight-bearing position. Time to each behavioral endpoint was recorded from the start of the d-amphetamine or vehicle infusion.

### Surgical Placement of Intracranial Electrodes

Neurophysiological recordings were taken from a group of three additional rats implanted with intracranial tetrodes and extradural electrodes to record local field potentials (LFP) and the electroencephalogram (EEG), respectively. Electrode placement was performed as previously described (Guidera et al., 2017). Briefly, animals were anesthetized with 2–3% isoflurane, and craniotomies were performed. Extradural EEG wires were placed above the prefrontal cortex (AP: +3.0 mm anterior-posterior (AP), medial-lateral (ML): −2.5 mm), the parietal cortex (AP: −6.0 mm, ML: −4.5 mm), and the cerebellar cortex (AP: −10.5 mm, ML: 0.0 mm). For EEG electrodes, a 3-cm insulated stainless-steel wire (#791400 steel, A-M Systems, Carlsborg, WA) with the coating removed at the tip was hooked under the skull and stabilized with dental cement. A ground screw (AP: −10.5 mm, ML: +2.4 mm) and five anchor screws (#51457, Stoelting, Wood Dale, IL) were also placed on the skull. The ground screw that was placed in the cerebellum, a region that is relatively quiescent during anesthesia experiments, provided a low impedance reference for electrical noise in the EIB-16 board. Tetrodes for LFP recordings were placed bilaterally in the prelimbic cortex (AP: +3.5 mm, ML: ± 0.8 mm, dorsal-ventral (DV): −3.8 mm) with a reference in the corpus callosum white matter (AP: 0.0 mm, ML: +1.6 mm, DV: −3.5 mm). This location was selected because it corresponds to the dorsal medial prefrontal and anterior cingulate cortices in humans, a location often recorded from in human EEG studies of anesthesia (Flores et al., 2017). For LFP recordings, tungsten tetrodes (0.001 inch diameter 99.95% tungsten wire, California Fine Wire Company, Grover Beach, CA) dipped in an organic dye, 1,1’-Dioctadecyl-3,3,3’,3’-Tetramethylindocarbocyanine Perchlorate (“DiI”; DiIC18(3)) (Vybrant DiI Cell-Labeling Solution, Thermo Fisher Scientific, Waltham, MA) were inserted at the above coordinates. While tetrodes are often used for recording single unit spiking, the use of tetrodes here was due their good signal recording and due to the stiffer durability of a tetrode compared to a single tungsten wire that bends easily when it is inserted into the tissue. Tetrodes were attached to the EIB-16 board with gold pins. At the end of the experiments, correct tetrode placement was confirmed with histological analysis (see Supplementary Figure S1). Electromyography (EMG) wires (#793500 steel, A-M Systems) were inserted bilaterally in the trapezius muscles. Electrode wires were fastened to an EIB-16 board (Neuralynx, Bozeman, MT) using gold pins (EIB Large Pins, Neuralynx, Bozeman, MT) and the board was secured above the skull with dental cement (Cat#51458, Stoelting, Wood Dale, IL). Throughout the surgery, the animal’s body temperature was maintained between 36.5–37.5°C, and heart rate and SpO_2_ were continuously monitored with a pulse oximeter placed on the hind paw. Following surgery, animals received 4 mg/kg of ketoprofen (Zoetis US, Parsippany, NJ) subcutaneously for analgesia on postoperative days 0–2. Animals recovered for at least 7 days before undergoing experiments.

### Local Field Potential Recordings and Analysis

Rats with LFP, EEG, and EMG electrodes underwent the same protocol for the administration of fentanyl and d-amphetamine as the blood gas and behavioral studies. Each rat received fentanyl (55 µg/kg, IV, over 15 min) and then either saline or d-amphetamine (3 mg/kg, IV, over 2 min). Rats received d-amphetamine and saline on separate days. Prior to fentanyl, 15–20 min of baseline recordings were taken in the awake state before fentanyl infusions were started. After the saline/d-amphetamine infusion, recordings were taken until 20–30 min after the return of righting. LFP recordings from the prelimbic cortex were digitally referenced to a tetrode placed in the corpus callosum with an external reference to the stainless steel screw placed in the skull over the cerebellum. Signals were continuously recorded with an Omniplex Neural Data Acquisition System (Plexon Inc, Dallas, TX). Analog signals were amplified with a 1x gain 16-channel headstage (HST/16o25-GEN2-18P-2GP-G1, Plexon), digitized at a sampling rate of 40 kHz with a Plexon MiniDigiAmp, digitally filtered (Bessel, four poles, 200 Hz cutoff) and down sampled to 1 kHz in the OmniPlex Server. Recordings were then analyzed using the Plexon OmniPlex and MAP Offline SDK Bundle and custom code written in MATLAB R2018b (The Mathworks Inc., Natick, MA). Spectrograms were computed using the Chronux function “mtspectrumc” using a TW = 3 and five tapers with a 3-s sliding window. Power spectral density (PSD) plots were generated for 60-s windows at three time points (15 min into the baseline recording, at the end of the fentanyl infusion (t = −2 min), and 5 min after the start of the d-amphetamine/saline infusion). The median power values were plotted by frequency with 95% confidence intervals derived from 1000-fold bootstrapping (calculated using the MATLAB bootstrap function “bootci”).

### Statistical Analysis

Data are expressed as Mean ± SD. *p* < 0.05 was considered statistically significant. A two-way mixed factor ANOVA was used to evaluate each behavioral measure (time to first head movement, time to two paws down, time to ROR) as a function of sex and drug treatment (saline or d-amphetamine) with a Bonferroni’s multiple comparison post hoc test. Blood gas data was analyzed using a mixed model repeated measures two-way ANOVA (α = 0.05) because one set of blood gas values (one of five samples from one rat) was inadvertently missed. Where significance was confirmed, a post-hoc Sidak’s test for multiple comparisons was performed. All values are reported as mean ± SD unless otherwise noted. All statistical analysis was performed with Prism 8.0 (GraphPad Software Inc., La Jolla, CA).

## Results

### D-Amphetamine Hastens Return of Consciousness After Fentanyl

**FIGURE 2:**
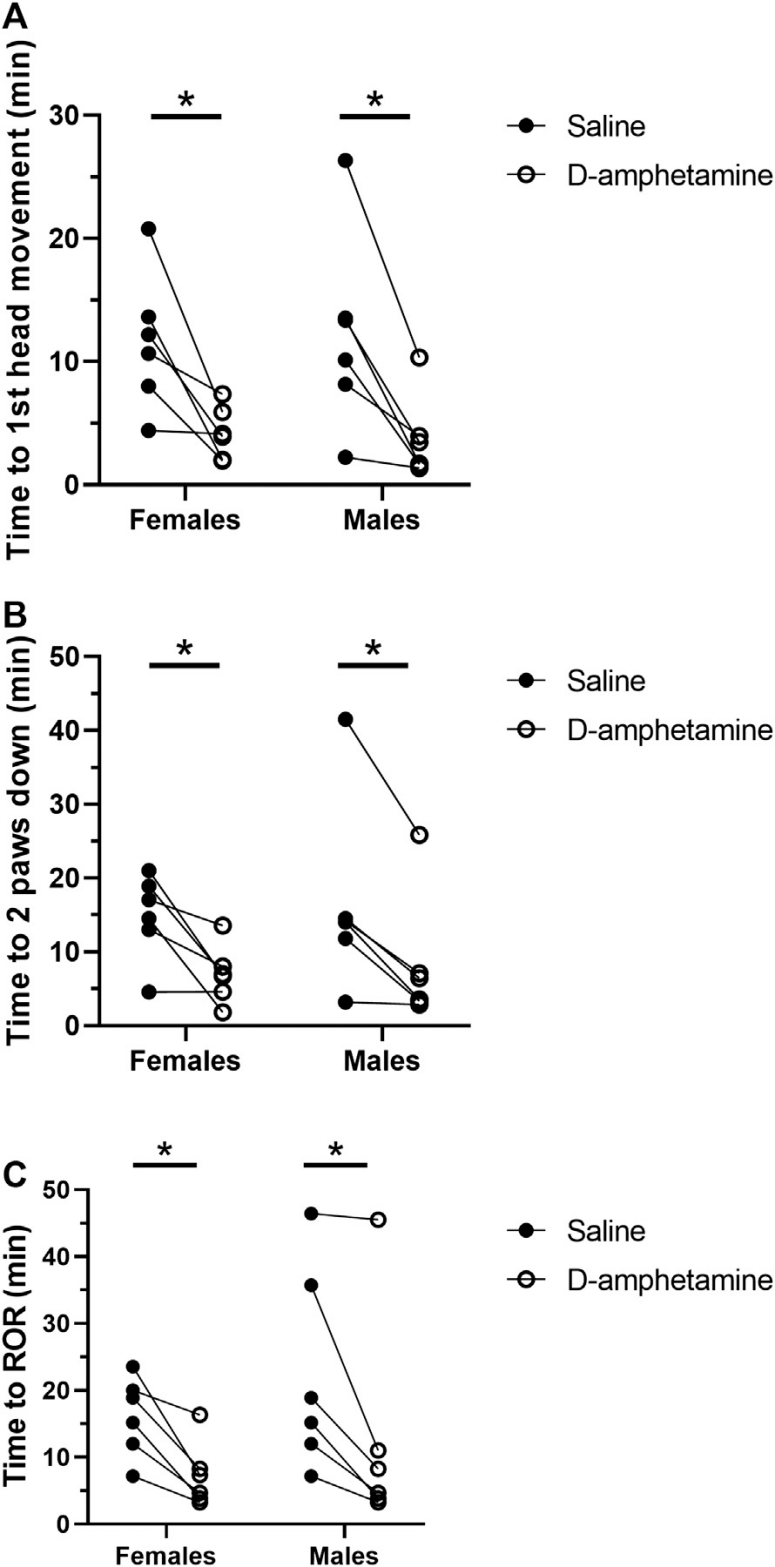
D-amphetamine (3 mg/kg, IV) hastens the recovery time for behavioral endpoints correlating with return of consciousness after fentanyl (55 µg/kg, IV). **(A)** Time to first head movement. A two-way mixed factor ANOVA and Bonferroni’s post hoc analysis revealed that d-amphetamine decreased time to first head movement significantly in females (t (6) = 3.370, *p* = 0.0142) and males (t (10) = 3.892, *p* = 0.0060). **(B)** Time to the return of two paws to the ground. A two-way mixed factor ANOVA and Bonferroni’s post hoc analysis revealed that d-amphetamine significantly decreased time to two paws down in females (t (10) = 3.553, *p* = 0.0105) and males (t (10) = 3.761, *p* = 0.0074). **(C)** Time to return of righting. A two-way mixed factor ANOVA and Bonferroni’s post hoc analysis revealed that d-amphetamine significantly decreased time to ROR in females (t (10) = 3.191, *p* = 0.0193) and males (t (10) = 3.536, *p* = 0.0108). *p < 0.05, n = 12 rats.

Behavioral scoring of the recovery period showed that intravenous d-amphetamine (3 mg/kg) hastened the return of consciousness after high-dose fentanyl (55 µg/kg) (Figure 2). The mean time to the first head movement was 66.6% faster when rats received d-amphetamine compared to saline (saline: 11.96 ± 6.59 min, d-amphetamine: 3.99 ± 2.72 min, n = 12). Next, the mean time to when the rats placed two paws down on the ground was 55.0% faster after d-amphetamine (saline: 16.83 ± 9.20 min, d-amphetamine: 7.58 ± 6.54 min, n = 12). Finally, the mean time to the return of the righting reflex was 36.6% faster when rats received d-amphetamine (saline: 19.24 ± 12.45 min, d-amphetamine: 12.19 ± 12.80 min, n = 12).

A two-way mixed factor ANOVA of time to first head movement as a function of sex and drug treatment (saline vs. d-amphetamine) showed no differences in sex on time to first head movement (F (1, 10) = 0.001577, *p* = 0.9691). The interaction of sex and drug was not significant (F (1, 10) = 0.1362, *p* = 0.7198). The main effect of drug was significant (F (1, 10) = 26.37, *p* = 0.0004). A Bonferroni’s multiple comparison test found that d-amphetamine in females decreased the time to first head movement by 7.393 ± 2.194 min (t (6) = 3.370, *p* = 0.0142). In males, d-amphetamine decreased the time to first head movement by 8.538 ± 2.194 min (t (10) = 3.892, *p* = 0.0060).

A two-way mixed factor ANOVA of the time to placing two paws down as a function of sex and drug treatment found no difference in sex (F (1, 10) = 0.09814, *p* = 0.7605). The interaction of sex and drug was not significant: F (1, 10) = 0.8864, *p* = 0.7198. The main effect of drug was significant: F (1, 10) = 26.75, *p* = 0.0004. A Bonferroni’s multiple comparison test found that d-amphetamine in females decreased the time to two paws down by 6.955 ± 7.887 min (t (10) = 3.553, *p* = 0.0105). In males, d-amphetamine decreased the time to first head movement by 8.198 ± 8.347 min (t (10) = 3.761, *p* = 0.0074).

A two-way mixed factor ANOVA of the time to return of righting (ROR) as a function of sex and drug treatment found no different between males and females (F (1, 10) = 0.8218, *p* = 0.3832). The main effect of drug was significant: F (1, 10) = 22.63, *p* = 0.0008. The interaction of sex and drug was not significant: F (1, 10) = 0.0596, *p* = 0.8121. A Bonferroni’s multiple comparison test found that d-amphetamine in females decreased the time to ROR by 8.857 ± 2.776 min (t (10) = 3.191, *p* = 0.0193). In males, d-amphetamine decreased the time to first head movement by 9.815 ± 2.776 (t (10) = 3.536, *p* = 0.0108).

The crossover design of this experiment was used to minimize potential effects of drug order on the behavioral measures between week one and week two. Although regularly used in pharmacology studies to minimize the effects of drug order, a two-way mixed factor ANOVA was performed on the ROR data to test whether time to ROR was vulnerable to an interaction effect between drug order and drug treatment. As expected, there was a significant effect of drug (saline vs. d-amphetamine) on time to ROR: F (1, 11) = 5.751, *p* = 0.0353. There was no significant effect of Drug Order on time to ROR: F (1, 11) = 1.386, *p =* 0.2639. Finally, there was no significant interaction between Drug Order and Drug effect on ROR: F (1, 11) = 0.3170, *p* = 0.5847. The lack of interaction validates the use of the crossover design for this protocol.

### D-Amphetamine Attenuates Respiratory Acidosis and Hypoxia After Fentanyl

**FIGURE 3:**
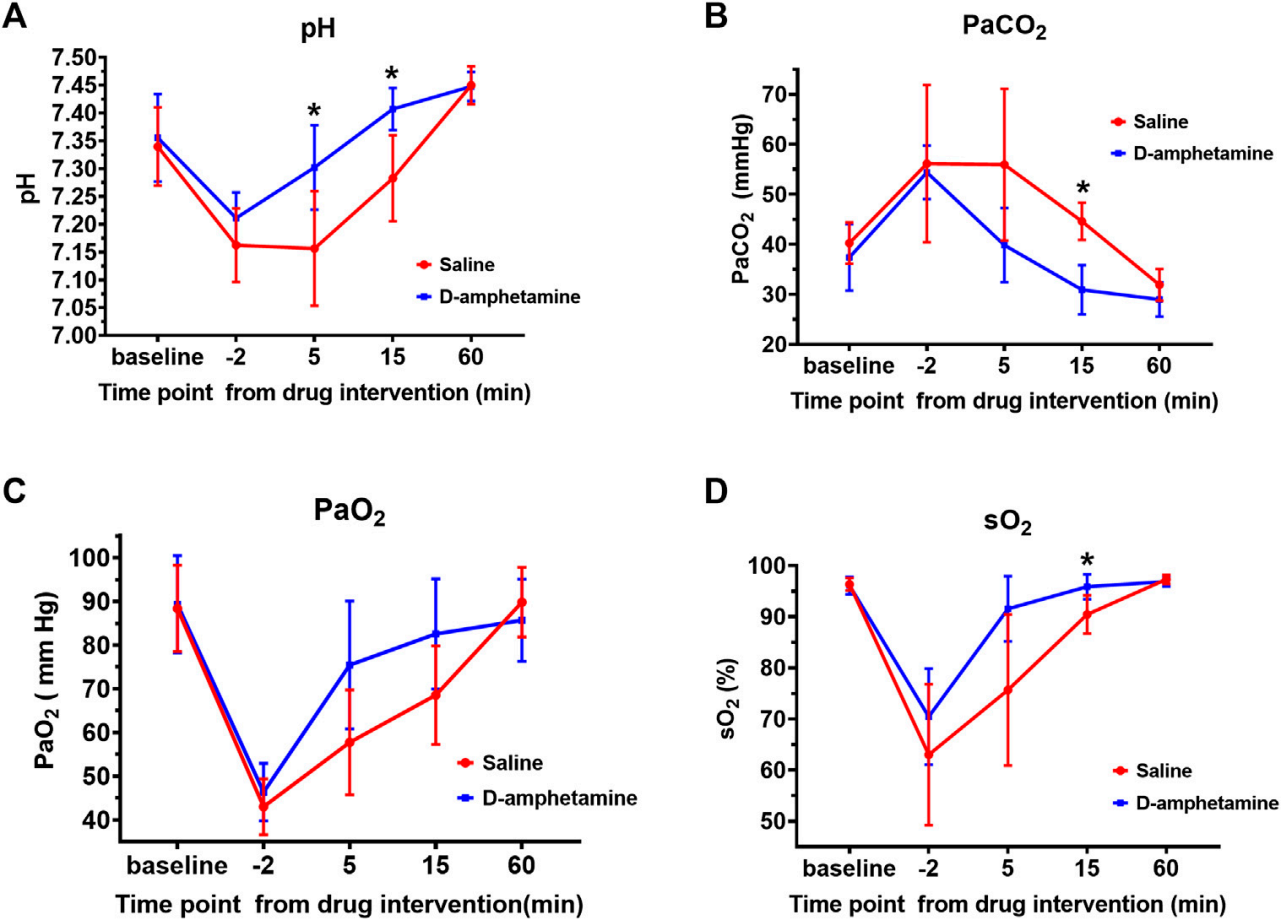
Arterial blood gas values after fentanyl (55 µg/kg, IV) followed by d-amphetamine (blue, 3 mg/kg, IV) or saline (red). Measures included **(A)** pH **(B)** the partial pressure of carbon dioxide (PaCO_2_) **(C)** the partial pressure of oxygen (PaO_2_), and **(D)** the percent oxygen saturation (sO_2_). Time points were defined from the start of the infusion of d-amphetamine or saline (t = 0). Blood was drawn for analysis at baseline, the end of the fentanyl infusion (t = −2 min), and at 5, 15, and 60 min after the d-amphetamine or saline infusion. *p < 0.05*,* significance was determined by a mixed model two-way, repeated measures ANOVA with a Sidak’s multiple comparisons post-hoc analysis. Points are mean ± SD from n = 9 rats.

Arterial blood gas analysis showed that the fentanyl infusion induced a marked respiratory acidosis that was alleviated by d-amphetamine (Figure 3). At the end of the fentanyl infusion (t = −2 min) there was an increase in PaCO_2_ (baseline: 38.8 ± 5.7 mmHg, t = −2 min: 55.3 ± 11.1 mmHg), decrease in pH (baseline: 7.35 ± 0.07, t = −2 min: 7.19 ± 0.06), and decrease in PaO_2_ (baseline: 88.8 ± 10.5 mmHg, t = −2 min: 44.7 ± 6.5 mmHg) across all groups relative to baseline. The administration of d-amphetamine led to faster recovery of arterial pH, PaCO_2_, PaO_2_, and sO_2_ values toward baseline levels at 5 and 15 min after the start of the d-amphetamine infusion (t = 0 min).

A two-way ANOVA with a Sidak’s post-hoc analysis was performed to test the effect of drug on arterial pH level over the course of the experiment. The main effect of drug was significant F (1, 16) = 6.349, *p* = 0.0228. The effect of time point was also significant: F (2.751, 43.32) = 104.9, *p* < 0.0001. The interaction of time point and drug was significant: F (4, 63) = 11.15, *p* < 0.0001. Specifically, after d-amphetamine the arterial pH (Figure 3A) was significantly higher at t = 5 min (saline: 7.16 ± 0.10, d-amphetamine: 7.30 ± 0.08, *p* = 0.0194, n = 9) and t = 15 min (saline: 7.28 ± 0.08, d-amphetamine: 7.41 ± 0.04, *p* = 0.0052, n = 9). At t = 60 min, all pH values had returned to slightly above baseline levels.

A two-way ANOVA with a Sidak’s post-hoc analysis was performed to determine the effect of drug on arterial PaCO_2_ over the course of the experiment. The main effect of drug was significant (F (1, 16) = 10.27, *p* = 0.0055), as was the effect of time point (F (2.123, 33.43) = 30.69, *p* < 0.0001). The interaction of Time and Drug was significant (F (4, 63) = 4.033, *p* = 0.0056). The PaCO_2_ (Figure 3B) was significantly decreased at t = 15 min after the administration of d-amphetamine compared to saline (saline: 44.6 ± 3.7 mmHg, d-amphetamine: 30.9 ± 4.9 mmHg, *p* < 0.0001, n = 9).

A two-way ANOVA with a Sidak’s post-hoc analysis was performed to determine the effect of drug on arterial PaO_2_ over the course of the experiment. The main effect of drug was significant (F (1, 18) = 6.868, *p* = 0.0044), as was the main effect of Time (F (2.922, 51.87) = 63.50, *p* < 0.0001). The interaction of Time and Drug was significant (F (4, 71) = 4.157, *p* = 0.0044). The PaO_2_ showed a trend toward faster recovery at t = 5 min after d-amphetamine administration but it did not reach statistical significance (saline: 57.7 ± 12.0 mmHg, d-amphetamine: 75.4 ± 14.6 mmHg, *p* = 0.0501, n = 9).

A two-way ANOVA with a Sidak’s post-hoc analysis was performed to determine the effect of drug on arterial sO_2_ over the course of the experiment. The main effect of drug was significant (F (1, 16) = 7.545, *p* = 0.0143). The main effect of time was also significant (F (1.859, 29.28) = 60.88, *p* < 0.0001). The interaction of time and drug was significant (F (4, 63) = 4.427, *p* = 0.0032). The sO_2_ was significantly higher at t = 15 min after d-amphetamine compared to saline (saline: 90.4 ± 3.7%, d-amphetamine: 95.9 ± 2.4%, *p* = 0.0132, N = 9). No significant differences were observed between male and female rats for any of the above data collected.

### D-Amphetamine Rapidly Restores Awake Neurophysiology After Fentanyl

**FIGURE 4:**
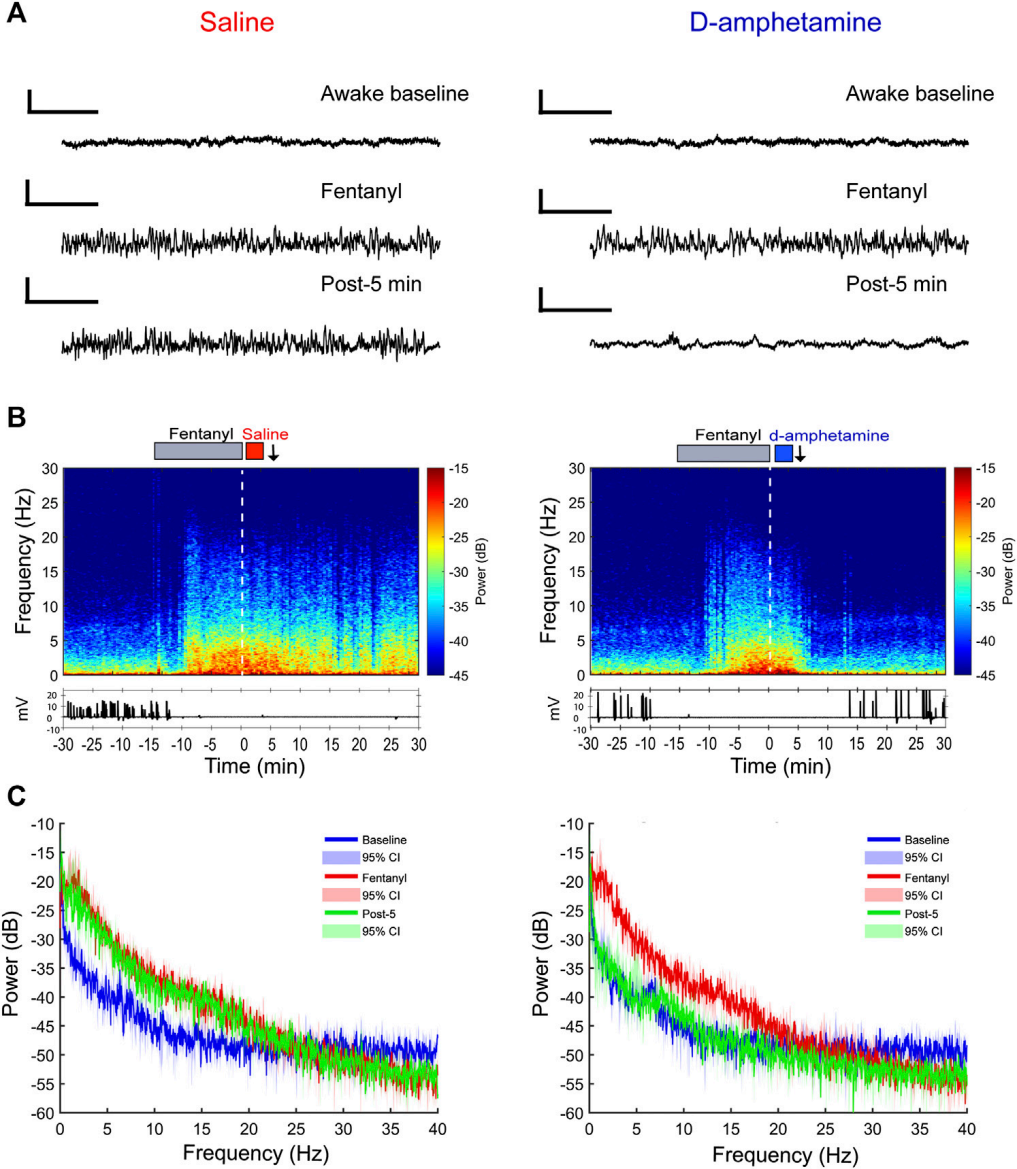
Prelimbic cortical local field potential recordings show a faster return of consciousness when rats receive d-amphetamine (3 mg/kg, IV) rather than saline after high-dose fentanyl (55 µg/kg, IV). **(A)** Sample raw waveforms from an individual animal during the awake baseline, during the fentanyl infusion, and 5 min after saline or d-amphetamine was administered. **(B)** Example spectrograms computed for an individual rat with the EMG waveform below. The gray bar above depicts the duration of the fentanyl infusion (15 min). The red bar indicates the saline infusion and the blue bar indicates the d-amphetamine infusion. The white dotted line represents t = 0 time point. The black arrows indicate the post-5-min time point at which raw waveforms and power spectral density (PSD) plots are shown above and below in parts **(A)** and **(C)**, respectively. **(C)** Group PSD plots computed from local field potential recordings taken during the awake baseline (blue), the end of the fentanyl infusion (red, t = −2 min), and 5 min after the start of the saline or d-amphetamine infusion (green, t = 5 min). Lines represent the median power and shaded areas represent the 95% confidence intervals. n = 3 rats.

In three additional rats, LFPs were recorded bilaterally from the prelimbic cortex during the administration of fentanyl and d-amphetamine (or saline) until return of righting occurred. Recordings from individual animals (Figure 4A) show that the characteristic fentanyl-induced LFP pattern remained beyond 10 min after saline administration, but that the baseline awake pattern was restored within 5 min after d-amphetamine administration. Spectrograms and corresponding EMG activity from individual rats (Figure 4B) demonstrate that fentanyl increased broadband power at frequencies <20 Hz (Figure 4B) that correlated with lack of movement. Even 20 min after saline, the increase in broadband power at frequencies <20 Hz and lack of EMG activity remained present, consistent with unconsciousness. However, after administration of d-amphetamine, broadband power at frequencies <20 Hz decreased rapidly in 1–2 min and returned to awake baseline levels. EMG activity also returned faster after d-amphetamine administration compared to saline. This effect was consistent across all three animals tested and individual spectrograms are included in Supplementary Figure S2. Power spectral analysis for the entire group (Figure 4C) revealed that 5 min after saline, the power spectrum remained largely unchanged from the end of the fentanyl infusion. However, 5 min after d-amphetamine, the power spectrum had largely returned to awake baseline levels.

## Discussion

This study tested the hypothesis that d-amphetamine hastens the recovery of consciousness and increases respiratory drive after high-dose fentanyl. Compared to saline controls, d-amphetamine decreased the time to first head movement and the return of the righting reflex, consistent with accelerated recovery of consciousness. Arterial blood gas analysis demonstrated that fentanyl induced profound respiratory acidosis, hypercapnia, and hypoxia, which were attenuated by d-amphetamine within 5 min of administration. LFP recordings from the prelimbic cortex showed that fentanyl generated broadband increases in power for frequencies <20 Hz, particularly in the delta band (<4 Hz), which is similar to anesthetic-induced loss of consciousness and non-rapid eye movement sleep (Brown et al., 2010). This is consistent with a previous report of spectrographic changes correlated with fentanyl-induced loss of consciousness and respiratory depression (Montandon and Horner, 2019). D-amphetamine caused these neurophysiological signatures of unconsciousness to return to awake baseline levels faster than in saline controls. Because d-amphetamine stimulates the dopamine system, which is also involved in movement, the LFP data demonstrates that d-amphetamine did not simply induce locomotion but also the recovery of awake neurophysiology. Together, these results show that d-amphetamine accelerates recovery from fentanyl-induced unconsciousness and respiratory depression.

Fentanyl has been shown to inhibit the release of arousal-promoting acetylcholine from pontine regions that modulate arousal, which likely contributes to loss of consciousness (Mortazavi et al., 1999). One likely mechanism by which d-amphetamine attenuates fentanyl-induced respiratory depression and unconsciousness is via its stimulatory effects on dopaminergic neurotransmission (Heal et al., 2013). Dopamine is involved in a variety of important brain functions including movement, reward, cognition, and wakefulness (Dahan et al., 2007; Beaulieu et al., 2015; Eban-Rothschild et al., 2016). Studies have shown that enhancing dopaminergic neurotransmission, specifically from the ventral tegmental area, restores consciousness in animals anesthetized by several different drugs including isoflurane, sevoflurane and propofol (Solt et al., 2011; Chemali et al., 2012; Taylor et al., 2013, 2016; Kenny et al., 2015). In addition, a D_1_ receptor antagonist blocked the arousal effects of d-amphetamine in the setting of sevoflurane anesthesia (Kenny et al., 2015). Furthermore, a D_1_ receptor agonist was found to alleviate fentanyl-induced respiratory depression in cats without attenuating analgesia (Lalley, 2004, 2005). Together, these findings suggest that the arousal-promoting and respiratory stimulant effects of d-amphetamine in the setting of fentanyl administration are likely mediated by the activation of D_1_ receptors.

D-amphetamine stimulates the direct release of monoamines into the synapse, which also raises the levels of serotonin in the brain (Heal et al., 2013). Neurons in the serotonergic raphe nucleus play an important role in respiration, airway control and other critical autonomic functions (Pilowsky, 2014). Previous animal studies of serotonin agonists have found that stimulating 5HT_1A_, 5HT_7_, and 5HT_4a_ receptors—all present on respiratory neurons in the rhythm-generating pre-Bӧtzinger complex—attenuated opioid-induced respiratory depression (Manzke et al., 2003; Meyer et al., 2006). Another important center in the brain containing 5HT_1A_ receptors is the medullary raphe nucleus of the brainstem (Heal et al., 2013). The abundance and diversity of serotonin receptors throughout the respiratory centers in the brain make it likely that serotonin is also involved in respiratory stimulation by d-amphetamine. However, human studies of serotonin agonists failed to show a significant effect in reversing morphine-induced respiratory depression (Lotsch et al., 2005; Oertel et al., 2007).

D-amphetamine also increases extracellular levels of norepinephrine, a neurotransmitter with a well-established role in promoting arousal (Carter et al., 2010). It is possible that norepinephrine also plays a role in the arousal-promoting actions of d-amphetamine. However, previous work found that atomoxetine, a selective norepinephrine reuptake inhibitor, did not induce behavioral arousal during continuous sevoflurane anesthesia (Kenny et al., 2015).

Experimental drugs that have been tested previously for the reversal of opioid-induced respiratory depression include the 5HT_4a_ receptor agonist BIMU-8 (Manzke et al., 2003), thyrotropin-releasing hormone (Horita et al., 1976), nicotinic acetylcholine receptor agonists (Ren et al., 2019), ampakines (Ren et al., 2006, 2009), microglia inhibitors (Hutchinson et al., 2008), and D_1_-receptor agonists (Lalley, 2004, 2005). However, many of these drugs have not been tested specifically with fentanyl, and most of them are not yet approved for human use. Previous animal studies testing d-amphetamine’s effects on morphine-induced analgesia suggest that a major advantage of d-amphetamine might be its ability to reverse negative side effects of opioid overdoses without decreasing analgesia (Dalal and Melzack, 1998). Measuring the effects of d-amphetamine on fentanyl-induced analgesia will be a crucial follow up experiment to the current study.

One limitation of this study was the use of a single d-amphetamine dose. The d-amphetamine dose was selected based on previous work that showed that d-amphetamine (0.3–3 mg/kg) hastened recovery from sevoflurane-induced unconsciousness in a dose-dependent manner (Kenny et al., 2015). Because the fentanyl dose chosen was very high and meant to mimic a fentanyl overdose, we chose the highest d-amphetamine dose (3 mg/kg) previously tested to maximize its effects when testing our hypothesis in this study. The effects of d-amphetamine in the absence of other drugs has been studied extensively in the context of neurophysiology (Lapish et al., 2012; Shen and Shi, 2020) and cardiorespiratory physiology (Mediavilla et al., 1979; Lin et al., 1980). However, future studies should include d-amphetamine dose-response studies to determine whether lower doses have differential effects on consciousness and respiration.

This study was not designed to test if d-amphetamine confers a survival benefit to rats after fentanyl overdose, but a future study might investigate this hypothesis. Because the mechanism of action of d-amphetamine is distinct from naloxone, future studies may also investigate whether d-amphetamine acts additively or synergistically with naloxone to treat respiratory depression. The pharmacokinetics of d-amphetamine elimination are slower than naloxone (U.S. Food and Drug Administration, 2019), making it less likely that repeated dosing will be necessary to maintain reversal of fentanyl-induced respiratory depression.

In summary, this study revealed that d-amphetamine accelerates the return of consciousness and attenuates respiratory depression induced by high-dose fentanyl in rats. Because d-amphetamine is already approved in oral form to treat Attention Deficit Hyperactivity Disorder and known to be safe in humans, it may provide a clinically useful alternative to naloxone with a distinct mechanism that does not target opioid receptors. More studies are needed to better understand the molecular and neural circuit mechanisms underlying the actions of d-amphetamine in the context of opioid reversal, which may lead to more targeted therapies for opioid overdose.

## Data Availability Statement

The raw data supporting the conclusions of this article will be made available by the authors, without undue reservation.

## Author Contributions

KS, OM, VA and JC contributed to the study concept and design. OM, EZ, VA, and RK contributed to the acquisition of data. EZ scored behavioral data. OM performed data analysis. OM and KS wrote the manuscript. All authors critically reviewed the content and approved the final version for publication.

## Funding

This research was supported in part by grants R01‐GM126155, P01‐GM118629, and F32‐GM137491 from the National Institutes of Health, Bethesda, Maryland, and by the 220020406 Scholar Award from the James S. McDonnell Foundation in Saint Louis, Missouri.

## Conflict of Interest

The authors declare that the research was conducted in the absence of any commercial or financial relationships that could be construed as a potential conflict of interest. KS is a consultant to Takeda Pharmaceuticals

## Abbreviations

**Abbreviations**: LFP: Local field potential; LOR: loss of righting; sO_2_: oxygen saturation; PACO_2_: partial pressure of carbon dioxide; PaO_2_, partial pressure of oxygen; ROR, return of righting.
