# Supplementary material for: D-Amphetamine Accelerates Recovery of Consciousness and Respiratory Drive After High-Dose Fentanyl in Rats
Source: Front Pharmacol. 2020 Nov 12;11:585356. doi: 10.3389/fphar.2020.585356 (PMC7793336; doi:10.3389/fphar.2020.585356)
Supplement: Supplementary file 1 [file datasheet1.pdf]

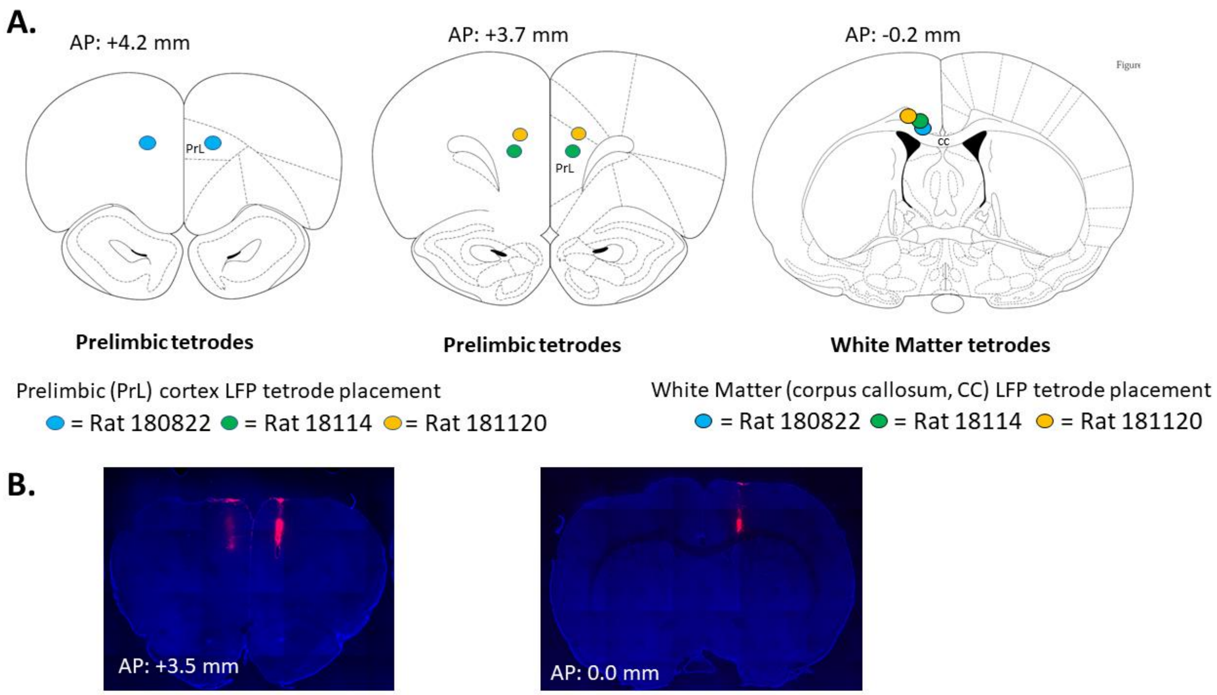

Figure S1. Histological confirmation of LFP tetrode placement in the prelimbic (PrL) cortex and corpus callosum (CC) white matter. The three rats (ID: 180822 (blue circle), 18114 (green circle), 181120 (yellow circle)) received tetrode placement bilaterally into the prelimbic cortex with a reference tetrode in the white matter. A) A schematic of the tetrode placement from the three individual rats shows correct placement of the tetrodes into the prelimbic cortex and corpus callosum. B) Representative images of the tetrode placement in the prelimbic cortex and corpus callosum using DiI (red) fluorescence show correct tetrode placement in rat 181120. These images were taken at 2.5X using a DAPI filter. Anterior-posterior (AP) distance from bregma is indicated on each slice image in millimeters.

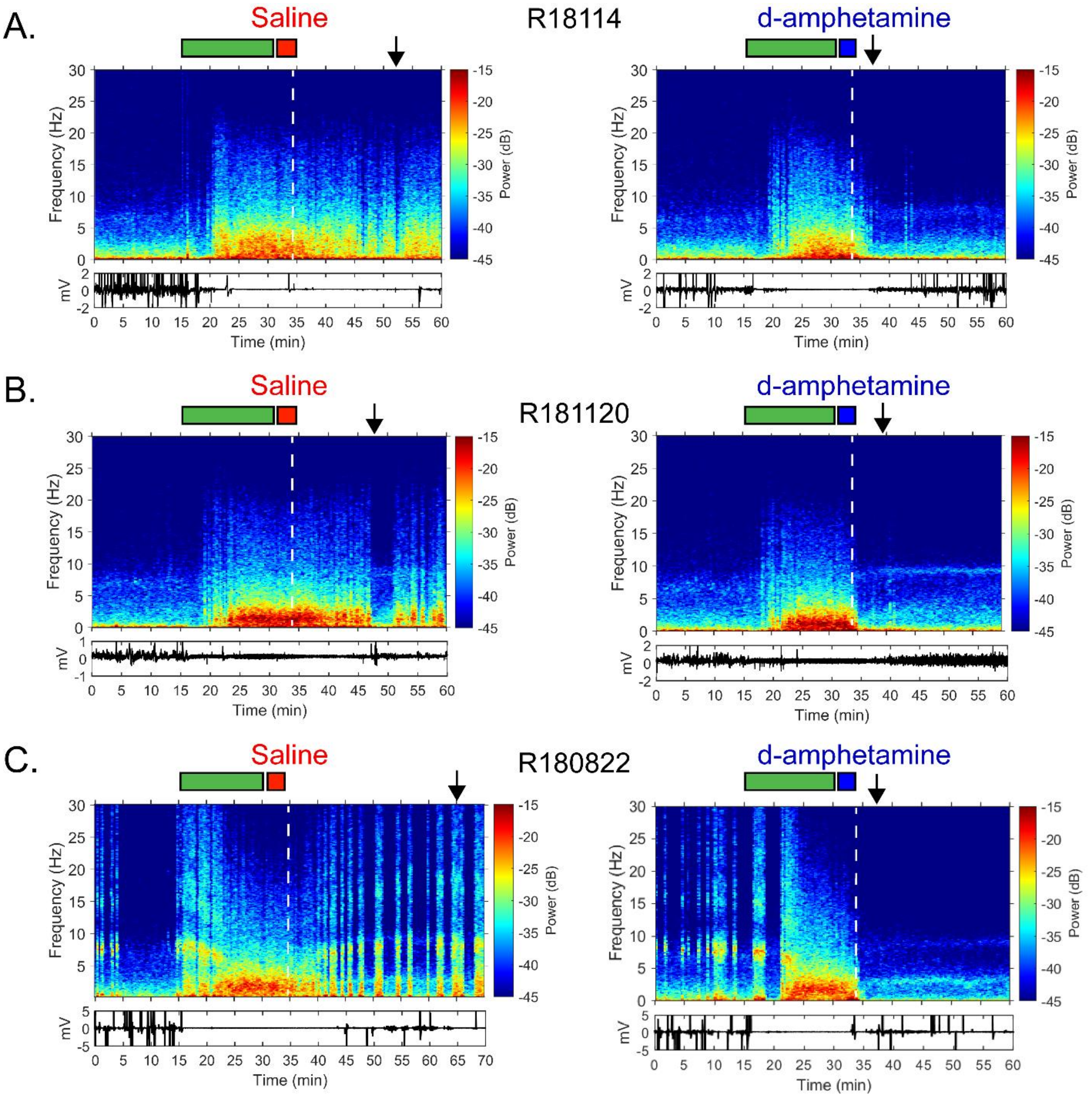

Figure S2. Individual spectrograms from the three rats recorded for LFPs from the right prelimbic cortex during high-dose fentanyl (55 µm/kg, IV) and either saline or d-amphetamine (3 mg/kg, IV) intervention. The three rats are: (A) R18114, (B) R181120, and (C) R180822. The green bar above the spectrogram represents the 15-minute fentanyl administration. The red bar represents the administration of saline and the blue bar of d-amphetamine. The dotted white line marks the end of the saline or d-amphetamine infusion. The black arrow represents the time of return of righting (ROR). The EMG signal for the corresponding time period is shown underneath each spectrogram.
